# A Versatile Aldehyde: Ferredoxin Oxidoreductase from the Organic Acid Reducing *Thermoanaerobacter* sp. Strain X514

**DOI:** 10.3390/ijms25021077

**Published:** 2024-01-16

**Authors:** Laura Sofie Nissen, Jimyung Moon, Lisa Hitschler, Mirko Basen

**Affiliations:** 1 Microbiology, Institute of Biological Sciences, University of Rostock, D-18059 Rostock, Germany; laura.nissen@uni-rostock.de; 2 Molecular Microbiology and Bioenergetics, Institute of Molecular Biosciences, Johann Wolfgang Goethe University Frankfurt/Main, 60438 Frankfurt/Main, Germany; moon@bio.uni-frankfurt.de (J.M.);; 3 Department of Maritime Systems, Interdisciplinary Faculty, University of Rostock, 18059 Rostock, Germany

**Keywords:** thermoanaerobacter, ethanol, aldehyde:ferredoxin oxidoreductase, AOR-ADH pathway, organic acid reduction

## Abstract

Aldehyde:ferredoxin oxidoreductases (AORs) have been isolated and biochemically-characterized from a handful of anaerobic or facultative aerobic archaea and bacteria. They catalyze the ferredoxin (Fd)-dependent oxidation of aldehydes to acids. Recently, the involvement of AOR in the reduction of organic acids to alcohols with electrons derived from sugar or synthesis gas was demonstrated, with alcohol dehydrogenases (ADHs) carrying out the reduction of the aldehyde to the alcohol (AOR-ADH pathway). Here, we describe the biochemical characterization of an AOR of the thermophilic fermentative bacterium *Thermoanaerobacter* sp. strain X514 (AOR_X514_). The putative *aor* gene (Teth514_1380) including a 6x-His-tag was introduced into the genome of the genetically-accessible, related species *Thermoanaerobacter kivui*. The protein was purified to apparent homogeneity, and indeed revealed AOR activity, as measured by acetaldehyde-dependent ferredoxin reduction. AOR_X514_ was active over a wide temperature (10 to 95 °C) and pH (5.5 to 11.5) range, utilized a wide variety of aldehydes (short and branched-chained, aliphatic, aromatic) and resembles archaeal *sensu stricto* AORs, as the protein is active in a homodimeric form. The successful, recombinant production of AOR_X514_ in a related, well-characterized and likewise strict anaerobe paves the road towards structure-function analyses of this enzyme and possibly similar oxygen-sensitive or W/Mo-dependent proteins in the future.

## 1. Introduction

Aldehyde ferredoxin oxidoreductases (AORs) catalyze the reversible reduction of carboxylic acids to aldehydes [1,2]. These enzymes belong to the DSMO-family of oxidoreductases and depend on a bis-metallopterin cofactor, with most AORs containing the biologically rare element tungsten (W) [3,4]. However, there are a few known AORs with a molybdenum (Mo) instead [5,6].

AORs occur either as homodimer [3], or as complexes consisting of three different subunits [7,8], but there are also monomeric [9] and homotrimeric [5] AORs described. While the monomeric and homodimeric AORs consist of one ~67 kDa subunit in α_2_ conformation, the more complex AORs consist of three different subunits with multiple alpha and beta subunits being connected by one gamma subunit. In AOR from *Moorella thermoacetica* (AOR_Mt_), α_3_β_3_γ seems to be the most prominent conformation [7], whereas in AOR from *Aromatoleum aromaticum* (AOR_Aa_) (αβ)_2_γ and (αβ)_3_γ conformations are found more often [8]. The formation of multimers was proposed for the former, as up to 1600 kDa complexes were found [7], and just recently the structure of the latter was examined and is was found that AOR_Aa_ forms long structures. These structures are similar to the spirosomes of AdhE [10] and even more similar to the recently described hydrogen-dependent carbon dioxide reductase (HDCR) from *Thermoanaerobacter kivui* [11]. The molecular function of these long filaments is not clear, but since the enzyme, as HDCR, accepts electrons from hydrogen or from an aldehyde, it may store and channel electrons along its backbone [8].

**Figure 1:**
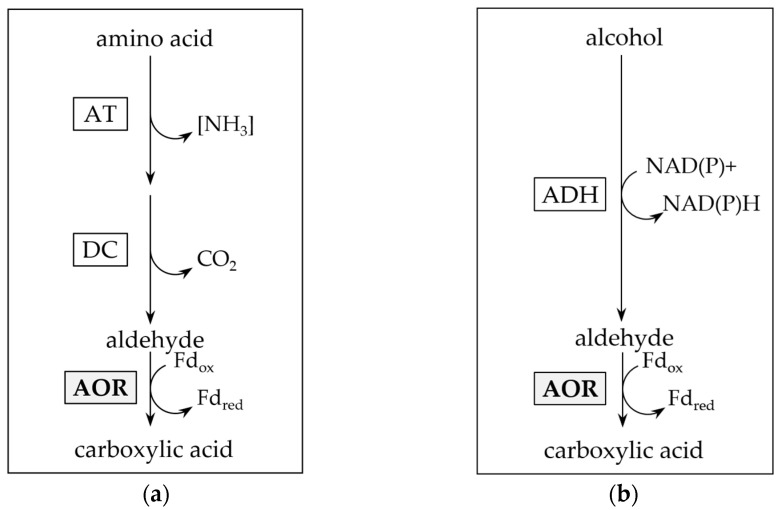
Putative function of aldehyde:ferredoxin oxidoreductase (AOR) in aldehyde detoxification. Reductive detoxification of accumulated aldehydes as product of amino acid ((**a**); after [17,18]) or alcohol catabolism ((**b**); after [14,19]). AT: aminotransferase; DC: decarboxylase; ADH: alcohol dehydrogenase.

AORs have been isolated from mesophilic, thermophilic and hyperthermophilic archaea and bacteria, and are mainly found in anaerobic microorganisms [1,2,5,6,9,12,13,14,15,16]. They are described as strict oxygen sensitive proteins, containing iron-sulfur clusters and a metallopterin cofactor. Two biological functions of AORs have been proposed. In the hyperthermophilic archaeon *Pyrococcus furiosus*, it was initially suggested that AOR removes toxic aldehydes, produced as by-products of peptide oxidation in the energy metabolism of the organism, by oxidation to their corresponding acids [2,13] (Figure 1a). A similar function has been proposed for the AOR of the mesophilic bacterium *Aromatoleum aromaticum* in phenylacetaldehyde oxidation [17]. This may be explained by the broad substrate spectrum of most AORs, including oxidizing activities towards aliphatic, aromatic and branched chain aldehydes [5,9,13,14,16].

**Figure 2:**
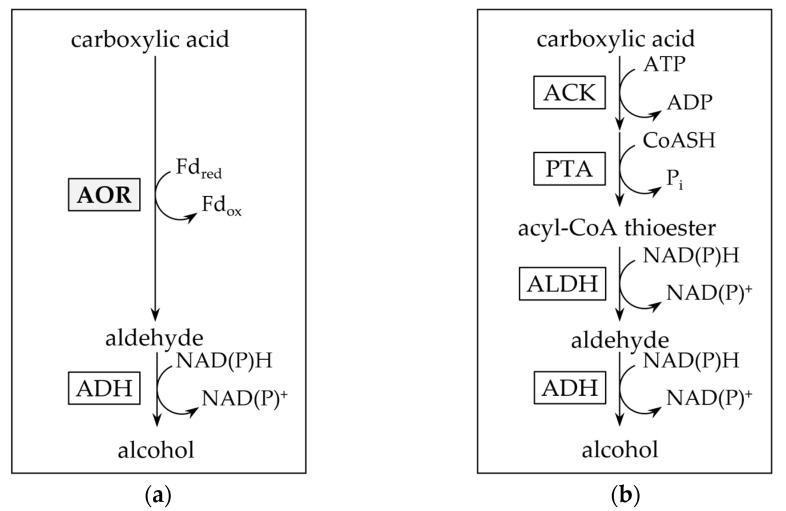
Pathways for enzymatic carboxylic acid reduction. (**a**) Aldehyde:ferredoxin oxidoreductase and alcohol dehydrogenase involving (AOR-ADH) pathway for direct reduction of carboxylic acids to alcohols. Note that the pathway also reduces acetate produced from pyruvate oxidation in sugar fermentation. (**b**) Activation of carboxylic acids through acetate kinase (ACK) and phosphotransacetylase (PTA) to their acyl-CoA thioesters, and subsequent reduction by aldehyde dehydrogenase (ALDH) and ADH.

A second function of AOR is its involvement in the reductive branch of the catabolism in fermentative bacteria and lithotrophic acetogens. Organic acid reduction, as observed e.g., in thermophilic *Moorella* and *Thermoanaerobacter* sp. may be carried out [20,21], on the one hand, with an acyl-CoA thioester as intermediate, which may subsequently be reduced by aldehyde dehydrogenase (ALDH) and alcohol dehydrogenase (ADH) (Figure 2b) [22]. On the other hand, in the first reaction, AOR may alternatively catalyze a direct reduction of the acid, e.g., acetic acid, to the aldehyde, e.g., acetaldehyde (Figure 2a). Since the standard redox potential of the pair acid/aldehyde is in the range of −520 to −560 mV [2], this requires the low redox potential electron carrier ferredoxin (Fd) as electron donor. Subsequently, the aldehyde is reduced to the corresponding alcohol by an (NADH or NADPH)-dependent alcohol dehydrogenase (ADH) [21]. This AOR-ADH pathway for alcohol production from carboxylic acids was first proposed in the late 1980s by Simon and colleagues, that found biochemical evidence for the involvement of a carboxylic acid reductase (later reclassified as AOR) in the thermophilic acetogenic bacterium *Moorella thermoacetica* [23]. The essential role of AOR in acid reduction was ultimately proven in an engineered strain of the hyperthermophilic archaeon *Pyrococcus furiosus* that contained a foreign ADH, and produced ethanol from sugars and alcohols from their corresponding acids [24]. More recently, AOR has been shown to be essential for ethanol formation from synthesis gas in *Clostridium autoethanogenum* or/and *Clostridium ljungdahlii* [25,26,27,28], with increasing evidence that this pathway may be widespread among sugar fermenting and gas (H_2_ or CO) oxidizing microorganisms [21].

The organotrophic thermophilic bacterium *Thermoanaerobacter* sp. strain X514 converts sugars to mostly ethanol and organic acids [20]. When supplied with external organic acids such as propionate or isobutyrate, it produced up to 43 mM of the corresponding alcohol [20]. The ability to reduce organic acids to alcohols with electrons derived from sugars is widespread among *Thermoanaerobacter* species [20,29]. Toward elucidating organic acid reduction in *Thermoanaerobacter* sp. strain X514, and in the absence of a genetic system, we purified the most abundant NADH- and NADPH-dependent alcohol dehydrogenases, the bifunctional aldehyde dehydrogenase (ALDH)/alcohol dehydrogenase, AdhE, and the secondary alcohol dehydrogenase AdhB, from the organism and biochemically characterized both, as well as two primary AdhA-type alcohol dehydrogenases [30]. Ethanol production from sugars and alcohol production from organic acids may proceed via reduction of acyl-CoA to aldehydes catalyzed by the ALDH AdhE, followed by reduction to the alcohol catalyzed by one of the ADHs, likely AdhB or the ADH activity of the bifunctional AdhE (Figure 2b) [22]. The strain however, phylogenetically groups with a few *Thermoanaerobacter* species that contain genes putatively encoding AORs, and it is among the group of strains that produce the highest titers of alcohols from acids, at rates comparable to those of *M. thermoacetica* [20].

Aldehyde-dependent reduction of benzyl viologen (BV), previously associated with AOR activity [5,14,16,31] was measured in the cell-free extract of *Thermoanaerobacter* sp. strain X514 [20], and the gene, *Teth514_1380* was transcribed at comparably high levels. This prompted us to propose that *Teth514_1380* likely encodes an active AOR. One the one hand, the enzyme may be involved in carboxylic acid reduction in *Thermoanaerobacter* sp. strain X514 [30]. One the other hand, the metabolic function of AOR may be the oxidative detoxification of aldehydes accumulating during alcohol or peptide catabolism [32], as *Thermoanaerobacter* sp. have been reported to oxidize amino acids [18].

Here, we took advantage of the genetic accessibility of the related, acetogenic *T. kivui* [33], that natively neither contains an *aor* nor produces alcohols, but converts H_2_ + CO_2_ and CO [34,35] at high rates to acetate. We expressed a *C*-terminally His-tagged version of *Teth514_1380* in *T. kivui*. The corresponding protein was characterized as a versatile AOR that may explain amino acid catabolism, and putatively, alcohol production in *Thermoanaerobacter sp. strain X514* and that paves the road towards future metabolic engineering efforts in *T. kivui* towards ethanol production.

## 2. Results and Discussion

### 2.1. Recombinant AOR-His Obtained from a Related Strain Is Active

To obtain enough protein for a biochemical characterization, we initially considered two options, the ‘native’ purification from *Thermoanaerobacter* sp. strain X514 or a recombinant expression in a suitable host. However, the expression in a standard host like *E. coli* is difficult due to the formation of the Fe_4_S_4_ and bis-W/Mo-opterin cofactors. Only two AORs have been produced recombinantly before, the *sensu stricto* AOR of *P. furiosus* was functionally produced in the extremely thermophilic anaerobic bacterium *Caldicellulosiruptor bescii* [36] and, recently, the novel type AOR of *A. aromaticum* was produced in the related strain *Aromatoleum evansii* [37]. Here, we took advantage of the recently described genetic system for the acetogenic bacterium *T. kivui*. This bacterium produces large quantities of the W-opterin and FeS-containing HDCR [11,38,39]; therefore, we assumed that it may be able to assemble these cofactors into a foreign protein, too. Proteins of *T. kivui* have been homologously-produced in *T. kivui,* either plasmid-based [33], or by integration into its genome [40]. A plasmid-based method to overproduce tagged proteins was established, with genes under the control of the strong constitutive promoter of the S-layer protein (P_Slp_) [41]. Here, we cloned the putative *aor (Teth514_1380)* gene with a C-terminal 6xHis-tag and controlled by P_Slp_ into plasmid pJM009 (Figure S1a). The plasmid also contained the *pyrE* gene, encoding a orotate phosphoribosyltranferase, which was transformed into the uracil auxotrophic strain *T. kivui* MB002 (old name TKV002; [33], and integrated into the genome (Figure S1b, strain MB014).

**Figure 3:**
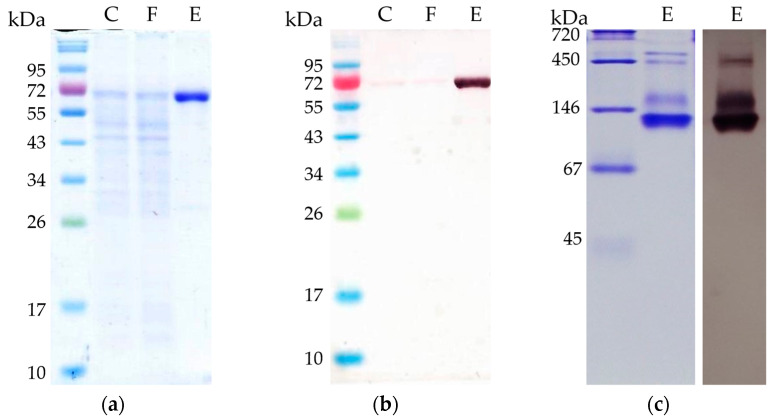
Separation of purified AOR_X514_ with C-terminal His-Tag by polyacrylamide gel electrophoresis (PAGE). C: 5 µg cell free MB014 extract before affinity chromatography; F: 5 µg flow-through of all proteins not binding to the His-tag column; E: 2 µg eluate containing AOR-His, eluted with 150 mM imidazole were analyzed (**a**) on a 10% SDS-polyacrylamide gel stained with Coomassie blue, (**b**) used for Western blot with specific anti-AOR antibodies and (**c**) on a 10% native polyacrylamide gel stained with Coomassie blue (**c**, left side) and used for Western blot with specific anti-AOR antibodies (**c**, right side). For SDS PAGE, NEB Color Prestained Protein Standard Broad Range (New England BioLabs) marker and for native gel Serva Native Marker (Serva) was loaded onto the gel.

*T. kivui* MB014 cells were grown, harvested and lysed as described in the method section of this article. Subsequently, the putative AOR_X514_ was purified by affinity chromatography to apparent homogeneity (Figure 3a) under strict anoxic conditions, since AORs have been reported to be extremely sensitive to oxygen [2,13]. Specific antibodies were used to verify the purification (Figure 3b). Using a protein standard, a subunit size of under 72 kDa was determined, which corresponds to the expected subunit size of AOR-His, 66.6 kDa. The purified protein is bound by the specific antibodies. Apart from AOR_Mt_ and AOR_Aa_, as described above, most of the characterized tungsten-dependent AORs consist of only one type of subunit, and form homodimers, except for AOR_Ea_, which most likely stays monomeric [9]. This homodimeric conformation can be found for hyperthermophilic archaeal AORs, such as those from *Pyrococcus furiosus*, *Thermococcus* strain ES-1, *Pyrococcus* strain ES-1 and *Pyrobaculum aerophilum* [3,13,15,32], but also for mesophilic bacteria such as AOR from *Clostridium formicoaceticum* and *Megalodesulfovibrio gigas* (formerly *Desulfovibrio gigas*) [12,14].

To elucidate the native composition, we ran a native PAGE (Figure 3c) and observed the most prominent protein with a measured size between 105 and 124 kDa. This corresponds to the expected size of an AOR-His homodimer (134 kDa). In addition, there are bigger proteins observed (between 290 and 520 kDa), which may be AOR_X514_ forming complexes. The formation of complexes has been described for other AORs, for example for AOR from *Clostridium formicoaceticum* (AOR_Cf_), where complexes of 240 kDa can be found in highly concentrated samples [12] and even bigger complexes of up to 1600 kDa were shown for AOR from *Moorella thermoacetica* (AOR_Mt_) [7]. And just recently, the formation of nanowires and thereby the formation of large complexes has also been demonstrated for AOR_Aa_ [8] as well.

Initially, we determined that the protein is an aldehyde oxidoreductase as it catalyzed the acetaldehyde-dependent reduction of benzyl viologen (BV) as an artificial electron acceptor, matching the activity in the cell free extract reported before [20]. Unfortunately, there was no acetaldehyde oxidation measurable in cell free extracts of *T. kivui* MB014 cells, and the enzyme activity of the purified protein was low, compared to previously purified AORs (less than 0.5 U mg^−1^, one unit (U) corresponding to 1 µmol acetaldehyde oxidized per minute [16,36,37], therefore we tested whether it was possible to improve enzyme activity, by adding different supplements to the growing *T. kivui* cells and testing different assay conditions.

### 2.2. AOR-His Activity Can Be Improved by Media Optimization

Most of the previously characterized AORs are W-containing enzymes and the increase of AOR activity through the addition of tungsten to the growing cells has been described previously [1], while molybdenum (Mo) is the antagonist for tungsten (W) [32]. The standard *T. kivui* [33] media contains 12 nM W and 490 nM Mo, i.e., about 40-times more Mo than W was supplied. Moreover, the *T. kivui* cell contains vast amounts of HDCR, which comprises one FeS cluster and one W per formate dehydrogenase subunit [11].

To determine whether the aldehyde oxidoreductase is W dependent and whether the activity can be improved by adding more W or less Mo, we supplied the growing *T. kivui* MB014 cells with different concentrations of W and Mo. The addition of W did not affect growth of *T. kivui* cells (Figure S2, Table S1), however, we saw a significant increase in AOR activity of the purified protein (Table 1). In this experiment, the standard amount of W (12 nM) led to an acetaldehyde oxidation activity of 0.04 U mg^−1,^ while the addition of 10-fold W (120 nM), increased the AOR activity by about 100-fold to 5.1 U mg^−1^. If 1.2 µM or 12 µM W was added, the activity was even higher (5.8 and 7.7 U mg^−1^, respectively), however, no further increase was observed with 120 µM or 1.2 mM. In contrast to W, the addition of Mo did influence growth of *T. kivui* and when adding 49 µM Mo (instead of 490 nM) the final optical density was much lower (Table S2) and AOR activity was reduced to 4% (Table 1), while omitting Mo did not improve the activity any further. Therefore, we routinely used the 1000-fold increased W concentration (12 µM) compared to the original *T. kivui* medium, with unchanged Mo concentration (490 nM), to optimize the amount of active protein, and achieved specific activities of up to 40 U mg^−1^.

As the purified enzyme contained only 3.2 Fe per subunit, compared to the expected 4 Fe, there may not have been enough iron supplied for the formation and integration of iron/sulfur clusters. Since a lot more sulfur than iron is supplied in the media (2 mM sulfate, 7.2 µM iron), we tested to supply to the growing cells an about 10-times higher iron concentration. Through this increase of iron in the media, AOR activity of the purified enzyme increased from 26 U mg^−1^ to 42 U mg^−1^ and the amount of Fe per subunit increased to 3.8 Fe per subunit (Table 1). In addition to W, Mo and Fe, we also tested other supplements such as different sulfur components, glucose, yeast extract, but only the addition of sodium sulfate and yeast extract increased the AOR activity (Table 1).

The effects of the different sulfur sources are relatively minor, likely due to the fact that *T. kivui* utilized the cysteine in the medium. Apart from sulfide, no inorganic sulfur source has been described to support growth of *T. kivui* [34].

There are multiple possible reasons as to why higher AOR activity is measured with higher tungsten concentration in the growth medium. *T. kivui* has tungsten enzymes such as the HDCR [11], therefore, it must have a mechanism to transport W into the cell, and a TupABC transporter is also encoded in the genome (TKV_c18720–18740). As the activity of AOR became eventually saturated at higher tungstate concentrations, it is a possibility that the transportation rate is too low to channel enough tungsten into the cell for the production of functional metabolic tungsten enzymes native in *T. kivui* and the additional, highly expressed AOR_X514_. A different explanation may be the cofactor synthesis. Unfortunately, there are not many studies focused on the maturation of the tungstopterin cofactor, but the tungstopterin in HDCR seems to be a tungstopterin guanine dinucleotide [11], while the tungstopterin of AOR_Pf_, for example, has no nucleotide appended [3]. These differences could potentially have an impact on the activity and further examination of the tungsten transporters and cofactor structure and its maturation in *T. kivui* may give more insights.

### 2.3. AOR_X514_ Is a Versatile Ferredoxin-Dependent Aldehyde Oxidoreductase

**Figure 4:**
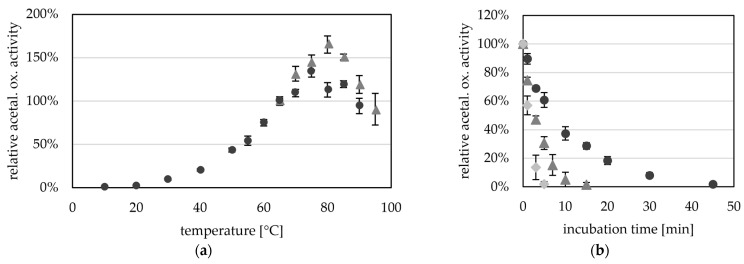
Temperature-dependent aldehyde oxidation activity of AOR_X514_. (**a**) AOR activity was measured between 10 °C and 90 °C (dark grey circles) and between 65 °C and 95 °C (grey triangles) as acetaldehyde-dependent reduction of BV (ε_600_ = 7.4 mM^−1^ cm^−1^). The reaction mixture contained in 50 mM TRIS, pH 7.5 with 1.2 mM BV, 12 µg (dark grey circles) or 15 µg (light grey squares) AOR_X514_ and 1.2 mM acetaldehyde. AOR-His from two independent purifications was used, the average represents four technical replicates. The specific activities were normalized to the mean value of the activity at 65 °C. (**b**) Thermostability of AOR at 65 °C (dark grey circles), 75 °C (grey triangles) and 85 °C (light grey squares). AOR activity was measured in 50 mM TRIS with 2.4 mM benzyl viologen, 14 µg AOR-His and 1.2 mM acetaldehyde at 65 °C. The average represents three to five experiments, activities were normalized to the enzyme activity before heat treatment.

Purified AOR_X514_ had a very wide temperature range, and we were able to measure activity between 10 °C (0.16 U mg^−1^) and 95 °C (8.2 U mg^−1^) with a temperature optimum at about 75 to 80 °C (Figure 4a). This temperature is slightly higher than the optimal growth temperature (60 °C) of *Thermoanaerobacter* sp. strain X514, however this effect has often been observed for enzymes from thermophiles, as for example observed for alcohol dehydrogenases AdhB and AdhA of the same organism [30], and also for bacterial AORs, such as AOR from *Megalodesulfovibrio gigas*, which has an optimum between 48 and 65 °C, while the organism grows around 30 °C [14]. AOR_X514_ is similar on amino acid level to AOR_Pf_ (61% identity); *P. furiosus* has optimal growth temperature of 100 °C, but the optimum of AOR_Pf_ is slightly lower (>90 °C) [2].

As bacterial AORs with the exception of AOR_Mt_ have been purified from mesophilic organisms, the optimal temperature (T_OPT_) of these AORs is generally lower than the ideal temperature of AOR_X514_ [4,12,14,16]. AOR_X514_ has therefore the highest T_OPT_ of all characterized, bacterial AORs, more comparable to the operating temperatures of archaeal AORs. An activation energy of 53 kJ mol^−1^ was calculated via Arrhenius plot for AOR_X514._ This value is lower than the activation energies calculated for the archaeal AORs from *Thermococcus* strain ES-1 and *Pyrobaculum aerophilum*, which have a high temperature optimum as well.

Although AOR_X514_ is more active at higher temperatures, AOR_X514_ has a relatively low thermostability and the half life time is only 7.3 min at 65 °C, 2.9 min at 75 °C and 1.4 min at 85 °C (Figure 4b). This is comparable to the half-life time of AOR_ES1_ which is 5 min at 70 °C and 1 min at 85 °C [13]. Considering the high optimal growth temperature of the organisms that both of these AORs are found in, the thermostability is very low. Also compared to, for example, AdhE and AdhB from *Thermoanaerobacter* sp. strain X514, which have stable activities at 65 °C for at least two or three hours, respectively [30].

It seems like AOR_X514_, and potentially even AORs in general, are extremely heat sensitive enzymes, which raises the question, whether they are more protected inside the cell, e.g., by a protein or by the osmolyte concentration, or whether this instability is the reason, AOR_X514_ is always highly expressed. Even though the activity was higher at a higher temperature we continued to measure the activity at 65 °C since the AOR is more stable at this temperature.

**Figure 5:**
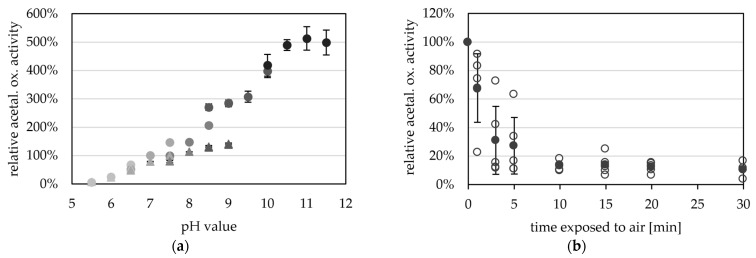
pH- and oxygen-dependent aldehyde oxidation activity of AOR_X514_. (**a**) AOR activity was measured between pH 5.5 and pH 11.5 with 0.2 mM benzyl viologen (BV, circles) and between pH 5.5 and pH 9.0 (triangles) with 2 mM BV at 65 °C, with 14 µg AOR-His and 1.2 mM acetaldehyde. The buffers used were at concentrations of 50 mM, MES (5.5, 6.0, 6.5), MOPS (6.5, 7.0, 7.5), TRIS (7.5, 8.0, 8.5, 9.0), CHES (8.5, 9.0, 9.5, 10.0) and CAPS (10.0, 10.5, 11.0, 11.5) were used (from lightest to darkest). The average represents four technical replicates. The specific activities were normalized to the mean value of 50 mM TRIS, pH7.5. (**b**) AOR_X514_ was exposed to air and the activity was measured regularly. Specific AOR activity was measured in 50 mM TRIS at pH 7.5 with 2.4 mM BV, 17 µg AOR_X514_ and 1.2 mM acetaldehyde at 65 °C. The average (filled circle) represents five separate experiments (empty circles), activities were normalized to the enzyme activity before air exposure.

Towards its pH optimum, acetaldehyde oxidation was observed at pH 5.5 (0.5 U mg^−1^) and increased activities up to pH 11.5 (36.3 U mg^−1^) (Figure 5a). A higher pH optimum for the aldehyde oxidation reaction has generally been reported for all AORs. However, the ideal pH for archaeal AORs from *Pyrococcus furiosus* and *Thermococcus* strain ES-1 is overall higher (pH 11 to 12) [2,13] than the ideal pH of bacterial AORs such as AOR from *Aromatoleum aromaticum* and *Clostridium formicoaceticum* (pH 8 to 10) [5,12,16].

The purified enzyme is extremely oxygen sensitive and has a half-life time of only 2.4 min when exposed to air at room temperature (Figure 5b). The oxygen sensitivity of purified AOR is reflected in the cell free extracts of *Thermoanaerobacter* sp. strain X514, or *T. kivui* MB014 (Figure S3), with half lives of 4 and 1.3 min in the CFE, respectively. This also reveals the necessity to purify the protein under anoxic conditions. Compared to other tungsten-dependent AORs, these values seem to be the norm, as AOR from *Thermococcus* strain ES-1, when exposed to air at room temperature, has a half-life time of 2 min in CFE and 1 min when purified [13]. AOR_Pf_ has only less than 20% activity left after 5 min exposure to air [2]. However, normally the sensitivity to oxygen is less extreme in the bacterial AORs, and AOR from *Megalodesulfovibrio gigas* has 20% activity left after 20 to 30 min [14], AOR from *Moorella thermoacetica* has still 60% activity left after 2 min [4] and AOR_Aa_ has a half life time of one hour, when exposed to air [16].

The protein, however, is very stable if kept at low temperatures in the absence of oxygen. AOR_X514_ can be stored for up to three weeks at 4 °C without loss of activity and for up to one year at −20 °C. In comparison to that AOR_X514_ only has 59% left after one week at room temperature, with only 2% activity left after one year at room temperature. As AOR_X514_ showed activity even at temperatures as low as 10 °C, it makes sense that even colder temperatures were required for long-term storage.

To test whether the position of the tag is of importance, we introduced an *N*-terminally His-tagged version of *Teth514_1380* (plasmid pMB009) (Figure S4), and subsequently treated the same way AOR-His was treated. The activity of His-AOR was a little higher (35.5 U mg^−1^) than that observed for AOR-His (25.1 U mg^−1^), but in a similar range, indicating that the position of the *tag* had a marginal influence.

**Figure 6:**
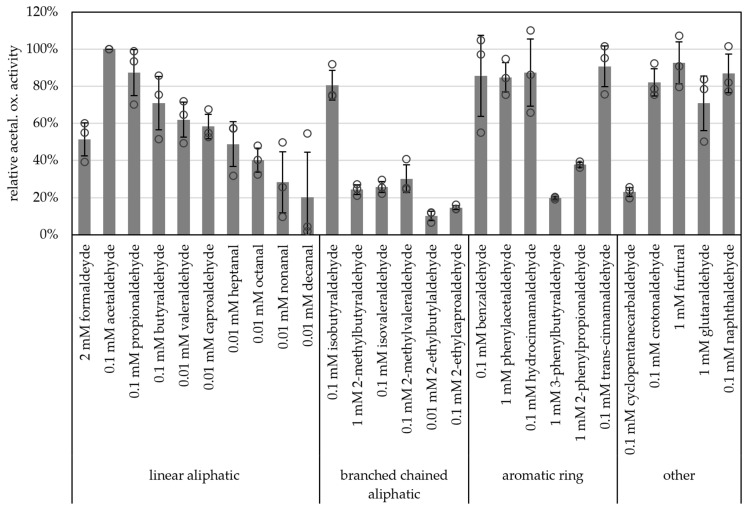
Substrate specificity of AOR-His. AOR activity was measured as aldehyde oxidation at 65 °C with 2 mM BV, 8 to 14 µg AOR-His and 2 mM, 1 mM, 0.1 mM or 0.01 mM aldehyde, as depicted. For each aldehyde the activity with 1 mM, 0.1 mM and 0.01 mM were tested, when the highest activity was with 1 mM, 2 mM aldehyde was tested additionally. The highest activity per aldehyde is represented in the figure. The average represents three separate experiments; activities were normalized to the enzyme activity with acetaldehyde.

Carboxylic acid reduction by microorganisms has gained attention in industry such as for bio-based polymers and production of alcohols as biofuels [42]. And, due to their broad substrate spectra, AORs have become more interesting for these applications. Along these lines, we tested a wide range of aldehydes as substrates. All of the supplied aldehydes, such as short-chained aliphatic (e.g., acetaldehyde, propionaldehyde) branched-chained aliphatic (e.g., isobutyraldehyde), aromatic (e.g., benzyaldehyde, phenylacetaldehyde, hydrocinnamaldehyde, trans-cinnamaldehyde) as well as unsaturated (crotonaldehyde), heterocyclic aldehydes (furfural) and naphthaldehyde were oxidized with comparably high specific activities (>80% of the activity with acetaldehyde, Figure 6 and Table S3). Although acetaldehyde was the best substrate tested, longer chained aldehydes such as nonanal and decanal were also oxidized (28% and 20% of activity towards acetaldehyde, respectively). Generally, AOR_X514_ showed a lower specific activity with branched-chained aldehydes than with the linear aldehyde, e.g., 71% butyraldehyde and 62% valeraldehyde versus 26% for isovaleraldehyde. For AOR_X514_ a wide variety of substrates were tested, also in comparison to other AORs, but overall, the high activity with short-chained linear aldehydes and short-chained aromatic aldehydes has also been described for other AORs, such as AOR from *Aromatoleum aromaticum* (AOR_Aa_) [16] and AOR from *Peptoclostridium acidaminophilum* (formerly *Eubacterium acidaminophilum*) [9]. Interestingly, furfural, which is toxic to most microorganisms and is a side product of pretreatment and hydrolysis or lignocellulosic feedstock and a problem in industrial wastewater [43,44], was oxidized.

As substrate inhibition has been described for AORs [1,2,9,13], the affinity and substrate inhibition for acetaldehyde was determined. The K_m_ for acetaldehyde was determined to be 8.4 ± 1.0 µM with a V_max_ of 15.5 ± 2.1 U mg^−1^ through Michaelis-Menten kinetic and for substrate inhibition, the k_i_ value was determined to be 8.7 ± 1.3 mM. Between about 0.03 and 3.0 mM the activity was constant (Figure S5a). In another experiment, a similar Km value of 9.7 µM was determined, but a higher V_max_ value of 38 U mg^−1^ (Table 2, Figure S5b). Compared to other AORs, this high affinity was expected as a K_m_ values for aldehydes between 10 and 200 µM had also been described for, for example AOR_Aa_ and AOR from *P. acidaminophilum* [9,16]. With longer chain length of the aldehydes, the V_max_ decreased, whereas the affinity stayed in the same range (Table 2). Based on a higher K_m_, formaldehyde might not be the preferred substrate for AOR_514_. Towards formaldehyde oxidation, other organisms such as *P. furiosus* have a separate formaldehyde oxidoreductase (FOR) [45,46].

When testing benzyl viologen and methyl viologen as artificial electron acceptors in acetaldehyde oxidation, a higher affinity for BV (K_m_: 2.1 mM; V_max_ 17.7 µmol min^−1^ mg^−1^) than for MV (K_m_: 19.2 mM; V_max_ 19.6 µmol min^−1^ mg^−1^) was measured (Table 2, Figures S6 and S7). Since MV has a lower redox potential (E = −450 mV; [47]) than BV (E = −374 mV; [47]), it might be less suitable to accept electrons during aldehyde oxidation. A similar difference between activity and affinity for BV and MV as electron acceptors has also been shown for AOR from *Megalodesulfovibrio gigas* [14] and for the AORs from *Thermococcus* strain ES-1 and *P. acidaminophilum* [9,13].

Fd is the putative native electron partner for all characterized AORs, since they are able to utilize BV, which is an indicator for Fd-dependence as archaeal AORs have been described to utilize both a viologen dye and Fd [32]. In these cases, ferredoxins native to the organism were purified and subsequently used for enzyme assays [2,13,15].

**Figure 7:**
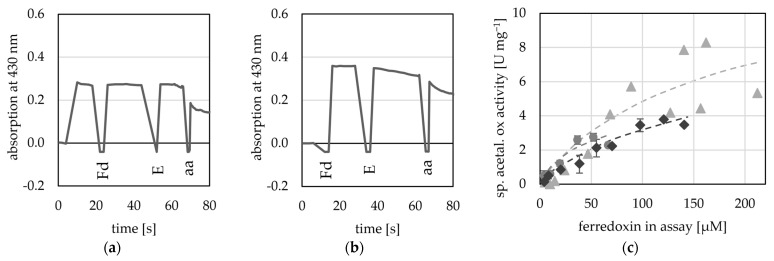
Ferredoxin-dependent AOR activity. AOR activity was measured at 65 °C as acetaldehyde-dependent Fd reduction at 430 nm in 50 mM TRIS buffer at pH 7.5. (**a**) 20 µM His- TKV_c16450 was used as electron acceptor (**b**) 20 µM His-TKV_c09620 was used as electron acceptor, 50 µg AOR_X514_ (E) were added and the reaction was started with the addition of 1 mM acetaldehyde (aa). (**c**) Between 4 to 212 µM His-TKV_c09620 were used as electron acceptor and 8 to 20 µg AOR-His, and 1 mM acetaldehyde. Three independent experiments with one to three technical replicates (grey circles, light grey triangles and dark grey diamonds).

Here, we used four potential Fds from *T. kivui* (TKV_c16450; TKV_c09620; TKV_c10420; TKV_c19530), as they can be overproduced in *T. kivui* and subsequently purified using a His-tag [48]. Of these four putative Fds, two were used by the AOR_X514_ for acetaldehyde oxidation (TKV_c16450; TKV_c09620) as electron acceptor (Figure 7), while the other two Fds (TKV_c10420; TKV_c19530) were not used (Figure S8). These findings correspond with the recently published characterization of these ferredoxins. Throughout a study carried out by Katsyv et al. (2023), it was found, that His- TKV_c16450 and His- TKV_c09620 are most likely ferredoxins involved in *T. kivui* metabolism [48]. The affinity for TKV_c09620 was higher than that for the artificial electron acceptors (50 to 200 µM) (Table 2), but since the absorption at 430 nm is saturated, when around 200 µM Fd are inserted into the assay, the absorption change cannot be measured reliably at higher concentrations and therefore no exact value was determined. Unlike the multimeric AORs from *M. thermoaceticum* [4] and *A. aromaticum* [16], AOR_X514_ neither used NAD^+^ nor NADP^+^ (Figure S9).

The characterization of the enzyme was carried out with assays in the direction of oxidation of the (intermediate) aldehyde. Assaying AOR in the direction of carboxylic acid reduction is difficult due to the low redox potential of the reaction and has only been demonstrated for few AORs [7,12,13]. Here, we showed that AOR indeed carried out MV^+^-dependent reduction of acetate, albeit only at a low rate and at pH 5.0 (Table 3). This is very characteristic since AOR is believed to utilize the undissociated acid as a substrate [12]. As aldehydes are extremely reactive, the addition of chemicals such as semicarbazide can be used to bind the produced aldehyde and thereby eliminating it from the reaction and reducing re/oxidation of the aldehyde [1,4,9]. Interestingly, the addition of semicarbazide to the acetate reduction assay here, led to the re/oxidation of MV^+^, which could afterwards not be re-reduced with dithionite and therefore this approach did not work. To eliminate acetaldehyde from the assay AdhE-His from *Thermoanaerobacter* sp. strain X514, purified from *E. coli* [30] was utilized, which, with the addition of NADH, can reduce acetaldehyde further to ethanol [1]. The elimination of acetaldehyde, by AdhE, led to a two-fold increase of acetate reduction activity from 7 mU mg^−1^ to 14 mU mg^−1^ (Figure S10). A similar coupled assay was also used to determine benzoate reduction of AOR_Aa_, using a benzyl alcohol dehydrogenase (BaDH) for elimination of benzaldehyde [37]. Neither in the uncoupled nor in the coupled assay, activity was measured with either one of the two purified Fds (His-TKV_c16450; His-TKV_c09620). The low specific activity towards the acid points towards a function in oxidative aldehyde removal, as suggested for AOR of *P. furiosus* before [2]. These aldehydes may be derived e.g., from peptide catabolism (the medium contains yeast extract). The in vitro rates, however, may not fully reflect the in vivo function, since AOR in organic acid reducing organisms such as *Moorella thermoacetica* also have a much lower K_m_ and V_max_ (~1000-fold and 5%, respectively) [1]. Hence, AOR may therefore also be involved in alcohol production from organic acids.

## 3. Materials and Methods

### 3.1. Cultivation of Thermoanaerobacter kivui Strains

*Thermoanaerobacter kivui* strains were routinely grown anaerobically in modified DSMZ171 media at 66 °C, as previously described [33]. The medium contained 50 mM Na_2_HPO_4_, 50 mM NaH_2_PO_4_, 1.3 mM K_2_HPO_4_, 1.6 mM KH_2_PO_4_, 7.6 mM NaCl, 5.9 mM NH_4_Cl, 1.7 mM (NH_4_)_2_SO_4_, 0.4 mM MgSO_4_, 7.2 µM FeSO_4_, 55.6 µM CaCl_2_, 1% trace element solution DSMZ141, 1% mL vitamin solution DSMZ141, 54 mM KHCO_3_, 2.8 mM cysteine-HCl and 2 g L^−1^ yeast extract (Carl Roth GmbH + Co. KG, Karlsruhe, Germany). If not stated otherwise, 12 µM tungsten (as tungstate; Na_2_WO_4_) was routinely added to the media for AOR-His and His-AOR purification. The medium was purged with Protadur C20 (80%/20% [*v*/*v*] N_2_/CO_2_) and then autoclaved. If not described otherwise, cells were grown with 25 mM glucose as substrate, added from a sterile, anoxic stock solution. Growth was monitored by measuring the OD at 600 nm.

*Thermoanaerobacter kivui* strains containing pMU131_His-TKV_c09620, pMU131_His-TKV_c16450, pMU131_His-TKV_c10420 and pMU131_His-TKV_c19530 for overproduction of *T. kivui* ferredoxins (Fd) were kindly provided by Prof. Volker Müller (Goethe University Frankfurt/Main). The construction of the plasmids for Fd overexpression is described elsewhere [48].

Cultivation of *Thermoanaerobacter* sp. strain X514 was performed as previously described [30].

### 3.2. Transformation of Thermoanaerobacter kivui

To generate *T. kivui* strains for production of AOR from *Thermoanaerobacter* sp. strain X514, plasmid pJM008 and pJM009 were constructed (Figure S11). Both plasmids were derived from plasmid pJM006 for overexpression of genes under the control of the S-layer promoter after integration into the genome region between Tkv_c24500 and Tkv_c24520 via homologous recombination [40]. The plasmid allows for selection using uracil auxotrophy, since it contains the *pyrE* gene under the gyrase promoter from *Thermoanaerobacter* sp. strain X514 [33]. For the generation of plasmids pJM008 and pJM009, the gene in pJM006 (*adhE*) was replaced with the *aor* gene from *Thermoanaerobacter* sp. strain X514 (*Teth514_1380*) in which 18 nucleotides encoding His_6_-tag (5′-CACCATCACCATCACCAT-3′) are fused either at the 5′ end (N-terminal His-tag; pJM008) or at the 3′ end of *aor* (C-terminal His-tag; pJM009). For the construction of pJM008, the His_6_-*aor* insert and pJM006 backbone were amplified by PCR using primers JM039 (5′-GAGGATTGACTGTATGCACCATCACCATCACCATTTTGGGTATGCCGGC-3′) and JM011 (5′-AAAAGCATGCTTCCCTCAAATTCCCAATTTTTGTAAAGTT-3′), and JM012 (5′-CTTTACAAAAATTGGGAATTTGAGGGAAGCATGCTTTTTAAAACAT-3′) and JM040 (5′-ATGGTGATGGTGATGGTGCATACAGTCAATCCTCCTCCTTGTA-3′), respectively. For the construction of pJM009, the *aor*-His_6_ insert and pJM006 backbone were amplified by PCR using primers JM010 (5′-CAAGGAGGAGGATTGACTGTATGTTTGGGTATGCCGG-3′) and JM041 (5′-GCTTCCCTCAATGGTGATGGTGATGGTGAATTCCCAATTTTTGTAAAGTTTCTTT-3′), and JM042 (5′-GGAATTCACCATCACCATCACCATTGAGGGAAGCATGCTTTTTAAAACA-3′) and JM013 (5′-CCGGCATACCCAAACATACAGTCAATCCTCCTCCTTGTATTT-3′), respectively. Then, the *aor* inserts were fused to the pJM006 backbone using Hifi assembly (New England Biolabs., Frankfurt am Main, Germany) to yield the plasmids pJM008 and pJM009. Subsequently, pJM008 and pJM009 were transformed into the ∆*pyrE* strain MB TKV002 [33] and integration of His_6_-*aor* or *aor*-His_6_ was carried out on agar plates with minimal medium without uracil. The integrated genome region of single colonies was analyzed by PCR with primers LH028 (5′-CAGGCTGTGATAATTTGAGAA-3′) and LH029 (5′-GGTCACGATTTAAAGGACTTA-3′) which bind at the 5′ end of the upstream flanking region (UFR) and at the 3′ end of the downstream flanking region (DFR) used for genome integration. Furthermore, the integrated region was verified by Sanger sequencing. After verification, the strain carrying His_6_-*aor* was named as TKV_MB009 and the strain *aor*-His_6_ as TKV_MB014.

### 3.3. Purification of His-Tagged Proteins from Thermoanaerobacter kivui

AOR-His purification was conducted under strictly anoxic conditions. Towards that, all material was placed inside an anoxic chamber (95% N_2_/5% H_2_ atmosphere; Coy Laboratory Products, Inc., Grass Lake, MI, USA) at least one day before use; all buffers were flushed with N_2_ and supplemented with 2 mM 1,4-dithioerythritol (DTE). *T. kivui* MB014 cells were grown in one to ten 2 L bottles with 1 L media and 25 mM glucose as substrate as described above. Cells were harvested in stationary phase (after 18–24 h, OD 0.5 to 1) and centrifuged for 20 min at 2700× *g* and 4 °C. The cells were washed with wash buffer (50 mM TRIS, 20 mM MgSO_4_, 150 mM NaCl, 20% [*v*/*v*] glycerol, pH 7.5, 2 mM DTE) and then resuspended in 20 mL start buffer (50 mM TRIS, 20 mM MgSO_4_, 150 mM NaCl, 10 mM imidazole, 20% [*v*/*v*] glycerol, pH 7.5, 2 mM DTE). Transfer to an anoxic chamber minimized contact of the intact cells to air during the harvest and washing steps. For this, the cell cultures were transferred into anoxic centrifuge bottles inside the anoxic chamber, the lids were closed, and then the closed bottles were taken out for centrifugation. After centrifugation, the centrifuge bottles containing the cell pellets were brought back into the anoxic chamber, and the media or buffer was removed, and the cells were resuspended in anoxic buffer. After addition of a spatula tip of DNase I and 0.5 mM phenylmethylsulfonyl fluoride (PMSF) the cells were transferred to a pressure cell under anoxic conditions and subsequently disrupted by a French Press at 110 MPa. The extract was collected in a degassed bottle filled with N_2_ to avoid contact with air. Cell debris was removed by centrifugation for 30 min at 37,000× *g* and 4 °C. The cell free extract was diluted to 52 mL. A 2 mL subsample was taken for enzyme activity, SDS-PAGE and Western blot, while the remaining 50 mL were used for the purification via affinity chromatography. AOR-His was purified using an Äkta Pure system (Cytiva, Marlborough, MI, USA) equipped with HisTrap HP 1 mL or 5 mL columns (Cytiva, Marlborough, MI, USA), placed in the anoxic chamber. Equilibration was performed with start buffer, while unspecific bound proteins were eluted with 2% elution buffer (50 mM TRIS, 20 mM MgSO_4_, 150 mM NaCl, 300 mM imidazole, 20% [*v*/*v*] glycerol, pH 7.5, 2 mM DTE), AOR-His was eluted with 50% elution buffer in start buffer, at a concentration of 150 mM imidazol.

His-AOR was purified from cell pellets of *T. kivui* MB009; grown in 1 L media and 25 mM glucose as substrate as described above. The ferredoxins His-TKV_c16450 and His-TKV_c09620 were purified from up to 8 L culture. Cultivation and production of the cell free extract was carried out under the same conditions as described above. His-AOR was purified using HisTrap HP 1 mL column (Cytiva, Marlborough, MI, USA) whereas TKV_c16450 and His-TKV_c09620 were purified using HisTrap HP 1 mL or HisTrap HP 5 mL column (Cytiva, Marlborough, MI, USA).

Protein concentrations were determined in a colorimetric assay according to Bradford [49], using ROTI^®^Nanoquant reagent (Carl Roth GmbH + Co. KG, Karlsruhe, Germany). Proteins were separated by SDS-PAGE with acrylamide concentrations between 10% and 16% [50]. For Western blot, the acrylamide gels were then transferred onto a nitrocellulose membrane [51].

The specific AOR-His antibodies were generated by immunization of a rabbit (Davids Biotechnologie, Regensburg, Germany) and used for immunological detection of the native protein in *Thermoanaerobacter* sp. strain X514 and control of successful purification. The size of AOR-His was determined by native PAGE under anoxic conditions. Towards that, polyacrylamide gel was prepared under air, then transferred into the anoxic chamber, and was stored in anoxic running buffer (25 mM TRIS, 96 mM glycine, pH 8.3) over night before use.

### 3.4. Enzyme Assays

Enzyme Assays were usually carried out under anaerobic conditions at 65 °C. The 1.4 mL quarz glass high performance cuvettes (Hellma, Müllheim, Germany) were closed with a rubber plug, flushed with N_2_ and then filled with 1 mL anaerobic TRIS-buffer (50 mM TRIS, 0.005% [*w*/*v*] resazurin, 2 mM DTE, pH 7.5) by injection through the stopper. Routinely, AOR activity was measured as acetaldehyde-dependent reduction of benzyl viologen at 600 nm (ε_600_ = 7.4 µmol min^−1^ mg^−1^) at 65 °C. For the reaction 2 mM benzyl viologen were used and between 2 and 100 µg protein, finally, the assay was started by the addition of 1 mM acetaldehyde from an anoxic stock solution. Activity was measured between pH 5.5 and 11.0 and between 10 °C and 95 °C, and routinely calculated as micromoles aldehyde oxidized per minute per milligram protein.

To determine the ferredoxin-dependency, benzyl viologen was replaced by 4 to 200 µM His-TKV_c16450, His-TKV_c09620, His-TKV_c10420 and His-TKV_c19530 from *T. kivui* [48]. The reduction of ferredoxin was measured at 430 nm, and the activity was calculated as micromoles aldehyde oxidized per minute per milligram protein, under the assumption that His-TKV_c16450 transports one electron and His-TKV_c09620 transports two electrons.

To determine acid-reduction of AOR, methyl viologen (MV; ε_600_ = 13.1 µmol min^−1^ mg^−1^) was used as electron donor. First, 10 to 20 µM MV in 50 mM anoxic TRIS buffer was reduced using sodium dithionite (from an anoxic 1 M stock solution). Subsequently, 24 µg AOR-His was added, and the reaction was started by adding 230 to 460 mM acetate. To determine acid reduction of AOR coupled to further reduction of the aldehyde to the alcohol, methyl viologen (MV) and NADH were used as electron donors for AOR and AdhE, respectively. First, 10 to 20 µM MV in 50 mM anoxic TRIS buffer was reduced using sodium dithionite (from an anoxic 1 M stock solution), then 0.5 mM NADH was added. Subsequently, 15 µg AdhE-His and afterwards 60 µg AOR-His was added, and the reaction was started by adding 450 mM acetate.

*E. coli* with the inducible plasmid pet21a::AdhE-His was grown in LB media with 100 µg mL^−1^ ampicillin. Production and purification of AdhE-His as previously described [30].

### 3.5. Quantification of Elements

Iron content of the purified enzyme was determined using a colorimetric assay with ferrozine (Fish, 1988) using ferrozine (Sigma-Aldrich, Merck KGaA, Darmstadt, Germany). The color change was measured at 593 nm. A standard of 0 to 200 µM ammonium iron sulfate hexahydrate was used to calculate the amount of iron in the samples.

The sulfur content of the purified enzyme was determined using a colorimetric assay [52] using N,N-dimethyl-p-phenylenediamine (DMPD) HCl. The sodium sulfide standard was made freshly every time from an anaerobic 1 mM stock solution and the samples were taken freshly from an anoxically stored solution. The change in color of DMPD was measured after centrifugation at 670 nm. A standard of 0 to 200 µM sodium sulfide was used to calculate the amount of sulfur in the samples.

For the quantification of tungsten and molybdenum in the purified AOR-His, the samples were sent to Spurenanalytisches Laboratorium Dr. Baumann (Maxhütte-Haidhof, Germany) for ICP-MS.

## 4. Conclusions

The results prove that TethX514_1380 is a *sensu stricto* dimeric archaeal type AOR, and may be acquired by horizontal gene transfer from archaea. Since the observed in vitro activities in the forward direction (acid reduction) are low, a major function of the enzyme may be the detoxification of aldehydes derived from peptide metabolism, as originally suggested for AOR of *P. furiosus*. Nonetheless, the enzyme may in be responsible for organic acid reduction in vivo, and explain at least part of the alcohol production from corresponding acids in *Thermoanaerobacter* sp. strain X514. The recombinant production of AOR paves the road to engineering in *T. kivui* for alcohol production and for structural studies of the protein using enzyme variants.

## Figures and Tables

**Table 1:** Effect of different components in the growth media on activity of purified AOR_X514_. The purification was routinely carried out with cells grown in media containing 12 µM W, 490 nM Mo, 11 µM Fe, 1 mM SO_4_, 0.2% (*w*/*v*) yeast (Roth) and 25 mM glucose. Changes in media composition and resulting relative specific activities are highlighted in bold in the table.

Tungsten	Molybdenum	Iron	Sulfur	Yeast Extract	Glucose	Relative Aldehyde Oxidation Activity
12 µM	490 nM	11 µM	2 mM SO_4_^2−^	0.2%	25 mM	**100%**
**1.2 nM**	490 nM	11 µM	2 mM SO_4_^2−^	0.2%	25 mM	**0.2%**
**12 nM**	490 nM	11 µM	2 mM SO_4_^2−^	0.2%	25 mM	**0.5%**
**120 nM**	490 nM	11 µM	2 mM SO_4_^2−^	0.2%	25 mM	**65%**
**1.2 µM**	490 nM	11 µM	2 mM SO_4_^2−^	0.2%	25 mM	**75%**
**120 µM**	490 nM	11 µM	2 mM SO_4_^2−^	0.2%	25 mM	**98%**
**1.2 mM**	490 nM	11 µM	2 mM SO_4_^2−^	0.2%	25 mM	**113%**
**120 nM**	**w/o**	11 µM	2 mM SO_4_^2−^	0.2%	25 mM	**79%**
**120 nM**	**4.9 µM**	11 µM	2 mM SO_4_^2−^	0.2%	25 mM	**63%**
**120 nM**	**49 µM**	11 µM	2 mM SO_4_^2−^	0.2%	25 mM	**4%**
12 µM	490 nM	**83 µM**	2 mM SO_4_^2−^	0.2%	25 mM	**160%**
12 µM	490 nM	11 µM	**11 mM SO_4_^−^**	0.2%	25 mM	**132%**
12 µM	490 nM	11 µM	+1 mM SO_3_^2−^ *	0.2%	25 mM	**66%**
12 µM	490 nM	11 µM	+1 mM S_2_O_4_^2−^ *	0.2%	25 mM	**95%**
12 µM	490 nM	11 µM	+1 mM S_2_O_3_^2−^ *	0.2%	25 mM	**55%**
12 µM	490 nM	11 µM	2 mM SO_4_^2−^	**1%**	25 mM	**191%**
12 µM	490 nM	11 µM	2 mM SO_4_^2−^	**1%**	**100 mM**	**164%**
12 µM	490 nM	11 µM	2 mM SO_4_^2−^	0.2%	**100 mM**	**53%**

* in addition to 2 mM SO_4_.

**Table 2:** Kinetic parameters of AOR-His. Values for K_m_ and V_max_ are given as mean values from two (*) or three different experiments.

	k_M_ [µM]	V_max_ [µmol min^−1^ mg^−1^]
BV	2122	17.7
MV	19230	19.6
His-TKV_c09620 (Fd)	~50 to 200	~8 to 19
Formaldehyde *	93.2	25.3
Acetaldehyde *	9.7	38.0
Propionaldehyde *	18.8	38.5
Butyraldehyde *	42.3	38.0
Valeraldehyde *	6.8	23.4
Capronaldehyde *	2.8	18.5
Heptanal *	7.4	15.3
Octanal *	17.1	17.8

**Table 3:** Comparison of AOR_X514_ acetaldehyde oxidation and acetate reduction activity with different redox Activities were normalized to the specific acetaldehyde oxidation activity with 2 mM oxidized BV. BV: benzyl viologen; MV: methyl viologen; Fd: ferredoxin.

	Redox Partner	Relative Specific AOR Activity
acetaldehyde oxidation	2 mM BV	100%
	19 mM MV	56%
	20 µM His-Fd09	4.4%
	20 µM His-Fd16	3.5%
acetate reduction	10 µM MV (uncoupled)	0.03%
	10 µM MV (coupled)	0.04%

## Data Availability

The datasets presented in this study can be found in the Supplementary Material.

## Supplementary Materials

The following supporting information can be downloaded at: https://www.mdpi.com/article/10.3390/ijms25021077/s1.
