# Supplementary material for: A Versatile Aldehyde: Ferredoxin Oxidoreductase from the Organic Acid Reducing Thermoanaerobacter sp. Strain X514
Source: Int J Mol Sci. 2024 Jan 16;25(2):1077. doi: 10.3390/ijms25021077 (PMC10816221; doi:10.3390/ijms25021077)
Supplement: Supplementary file 1 [file ijms-25-01077-s001.zip › Supplements_ijms-2752207_revised.pdf]

A versatile aldehyde:ferredoxin oxidoreductase from the organic acid reducing *Thermoanaerobacter* sp. strain X514

Laura Sofie Nissen, Jimyung Moon, Lisa Hitschler, Mirko Basen

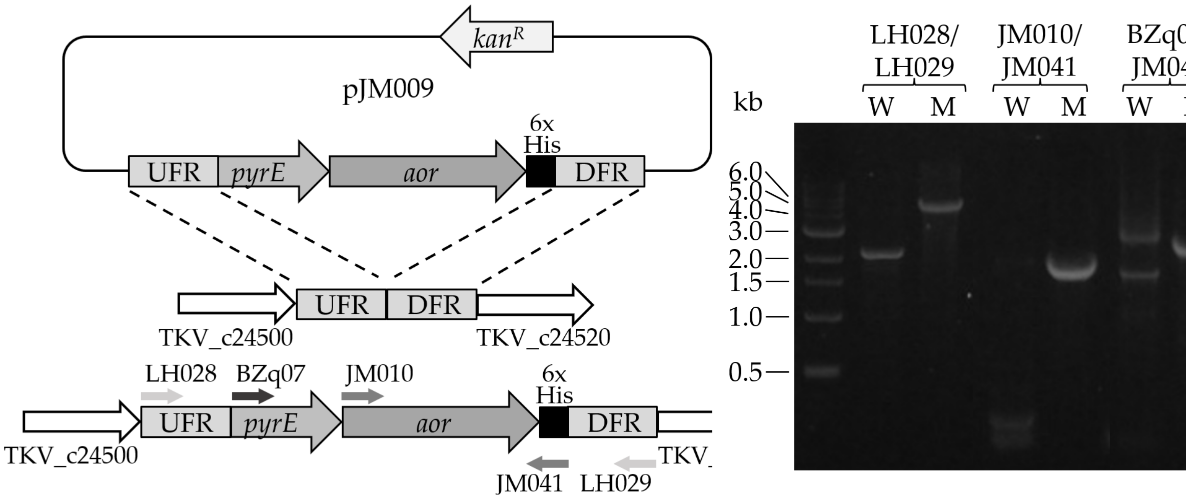

(**a**) (**b**)

Figure S1. Integration of *aor*-His (*Teth514_1380*) in *T. kivui*. (**a**) Schematic overview of *pyrE* and *aor* gene integration between TKV_c24500 and TKV_c24500. Primers used for generation and verification of the strain are annotated. (**b**) Agarose gel electrophoretic analysis of PCR products, with W indicating *T. kivui* wild type DSMZ 2030 DNA and M indicating the "mutant" *aor*+ strain *T. kivui* MB014 DNA as templates.

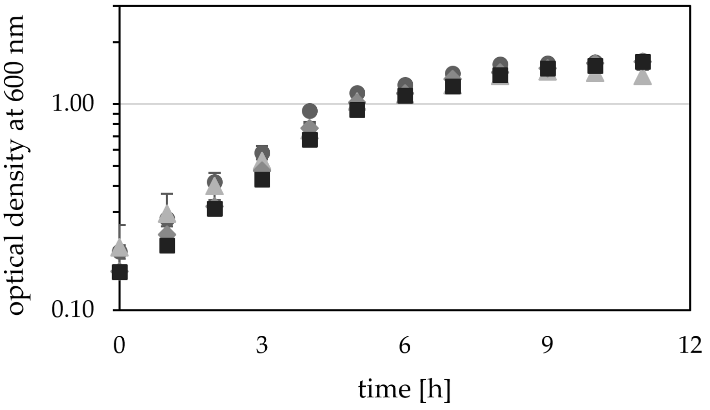

Figure S2. Effect of different tungsten concentrations in the growth medium on *T. kivui* growth. *T. kivui* was grown at 65 °C and optical density was determined hourly at 600 nm. Cells were grown with medium containing 12 nM tungsten (dark grey circles), 120 nM tungsten (light grey triangles), 1.2 µM tungsten (grey diamonds) or 12 µM tungsten (black squares). The average represents two biological replicates.

Table S1. Effect of different tungsten concentrations in the growth medium on *T. kivui* growth. *T. kivui* was grown at 65 °C and optical density was determined hourly at 600 nm. Maximal optical densities and growth rates were determined. The average represents two biological replicates.

	**maximal optical density**	**growth rate [h^{-1}]**
**12 nM W**	1.627 ± 0.004	0.353 ± 0.002
**120 nM W**	1.469 ± 0.027	0.322 ± 0.063
**1.2 µM W**	1.608 ± 0.007	0.370 ± 0.026
**12 µM W**	1.610 ± 0.012	0.378 ± 0.001

Table S2. Effect of different molybdenum concentrations in the growth medium on *T. kivui* growth. *T. kivui* was grown at 65 °C and optical density was determined at 600 nm after 18 h growth.

	**optical density after 18 h**
**49 nM Mo**	1.52
**490 nM Mo**	1.42
**4.9 µM Mo**	1.58
**49 µM Mo**	0.20

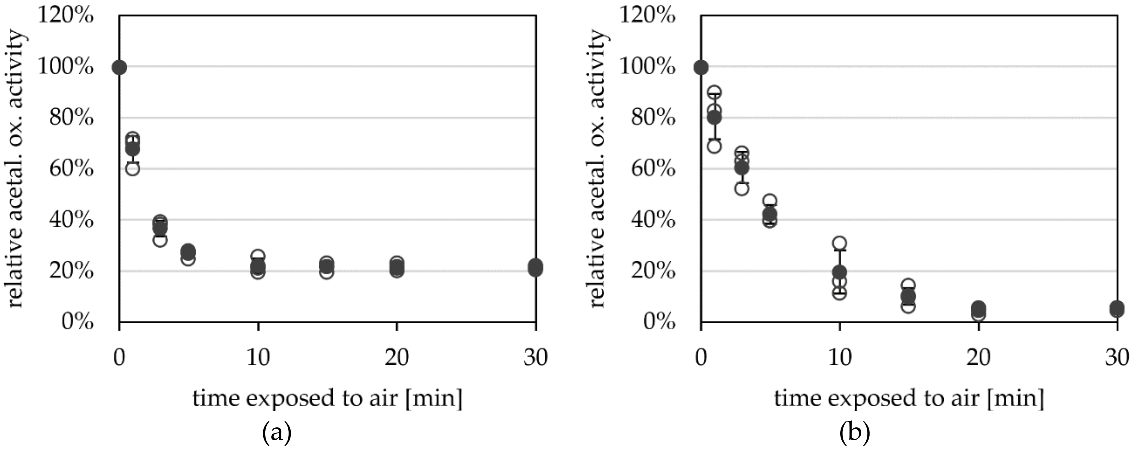

Figure S3. Influence of oxygen on AOR$_{X514}$ activity in cell free extract. (**a**) CFE of *T. kivui* MB014 was exposed to air and the activity was measured regularly. Specific AOR activity was measured in 50 mM TRIS at pH 7.5 with 2 mM BV, 100 µg CFE and 1 mM acetaldehyde at 65 °C. The average represents three separate experiments, activities were normalized to the enzyme activity before air exposure (**b**) CFE of *Thermoanaerobacter* sp. strain X514 was exposed to air and the activity was measured regularly. Specific AOR activity was measured in 50 mM TRIS at pH 7.5 with 2 mM BV, 103 µg CFE and 1 mM acetaldehyde at 65 °C. The average represents three separate experiments, activities were normalized to the enzyme activity before air exposure.

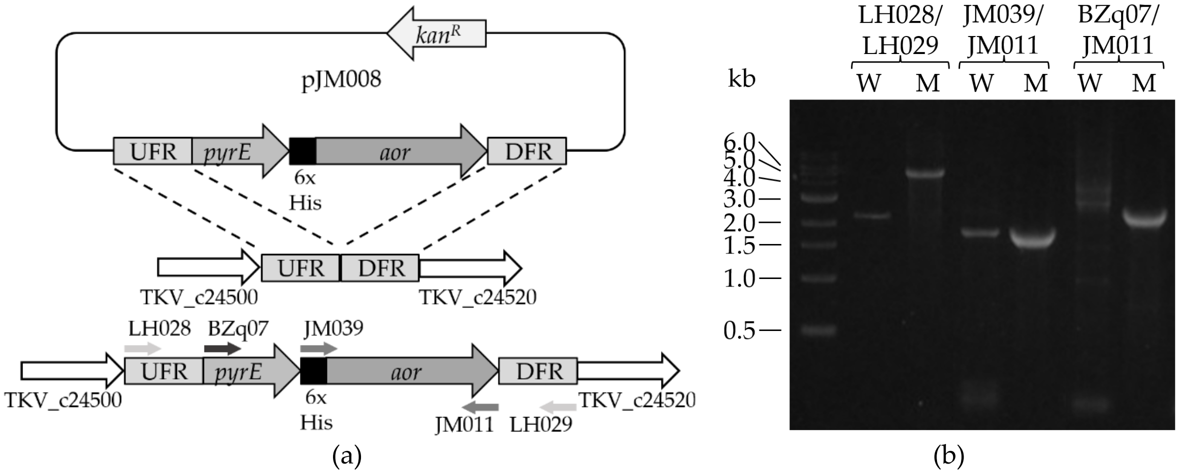

Figure S4. Integration of His-*aor* (*Teth514_1380*) in *T. kivui*. (**a**) Schematic overview of *pyrE* and *aor* gene integration between TKV_c24500 and TKV_c24500. Primers used for generation and verification of the strain are annotated. (**b**) Analysis of fragments on a 1 % agarose gel, with W indicating *T. kivui* wild type DSMZ2030 DNA and M indicating the mutant strain *T. kivui* MB009 DNA.

Table S3. Overview of the relative specific aldehyde oxidation activities of AOR_{X514} with different aldehydes. Activities were normalized to acetaldehyde oxidation activity.

Substrate	Relative specific aldehyde oxidation activity [%]	Standard derivation
2 mM formaldehyde	51%	9%
0.1 mM acetaldehyde	100%	0%
0.1 mM propionaldehyde	87%	12%
0.1 mM butyraldehyde	71%	14%
0.01 mM valeraldehyde	62%	9%
0.01 mM caproaldehyde	58%	7%
0.01 mM heptanal	49%	12%
0.01 mM octanal	40%	6%
0.01 mM nonanal	28%	17%
0.01 mM decanal	20%	24%
0.1 mM isobutyraldehyde	81%	8%
1 mM 2-methylbutyraldehyde	24%	3%
0.1 mM isovaleraldehyde	26%	3%
0.1 mM 2-methylvaleraldehyde	30%	7%
0.01 mM 2-ethylbutylaldehyde	10%	2%
0.1 mM 2-ethylcaproaldehyde	15%	1%
0.1 mM benzaldehyde	86%	22%
1 mM phenylacetaldehyde	85%	8%
0.1 mM hydrocinnamaldehyde	87%	18%
1 mM 3-phenylbutyraldehyde	20%	1%
1 mM 2-phenylpropionaldehyde	38%	1%
0.1 mM cyclopentanecarbaldehyde	23%	2%
0.1 mM crotonaldehyde	82%	7%
0.1 mM trans-cinnamaldehyde	91%	11%
1 mM furfural	93%	11%
1 mM glutaraldehyde	71%	15%
0.1 mM naphthaldehyde	87%	10%

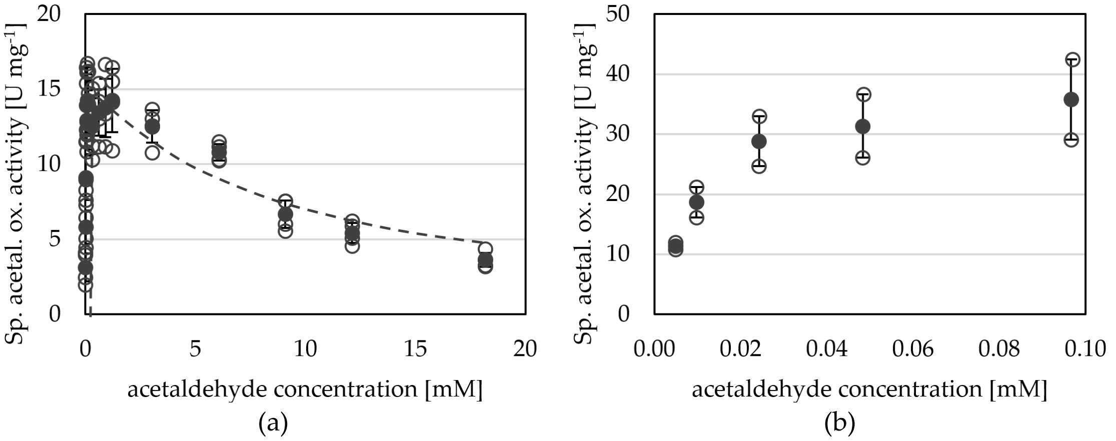

Figure S5. Influence of acetaldehyde concentration on AOR_{X514} activity. (**a**) Specific acetaldehyde oxidation activity was measured in 50 mM TRIS at pH 7.5 with 1 mM BV, 5 µg AOR-His and between 0.0025 and 18.2 mM acetaldehyde at 65 °C. The average represents four technical replicates. (**b**) Specific acetaldehyde oxidation activity was measured in 50 mM TRIS at pH 7.5 with 2 mM BV, 4 to 22 µg AOR-His and between 0.005 and 0.1 mM acetaldehyde at 65 °C. The average represents two technical replicates.

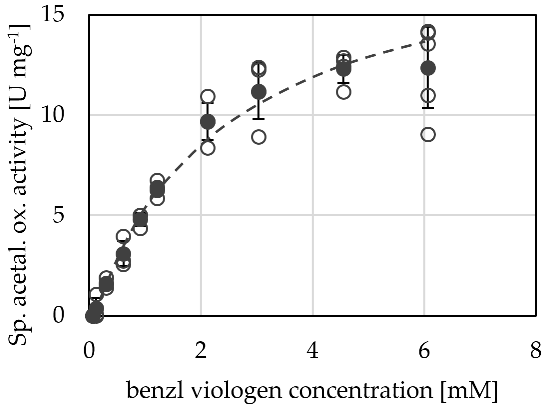

Figure S6. Influence of benzyl viologen concentration on AOR_{X514} activity. Specific acetaldehyde oxidation activity was measured in 50 mM TRIS at pH 7.5 with 0.06 to 6 mM BV, 6 µg AOR-His and 1.2 mM acetaldehyde at 65 °C. The average represents four technical replicates.

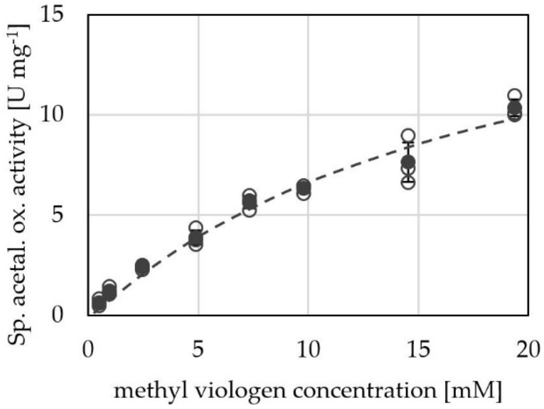

Figure S7. Influence of methyl viologen concentration on AOR_{X514} activity. Specific acetaldehyde oxidation activity was measured in 50 mM TRIS at pH 7.5 with 0.5 to 20 mM MV, 32 µg AOR-His and 1 mM acetaldehyde at 65 °C. The average represents four technical replicates.

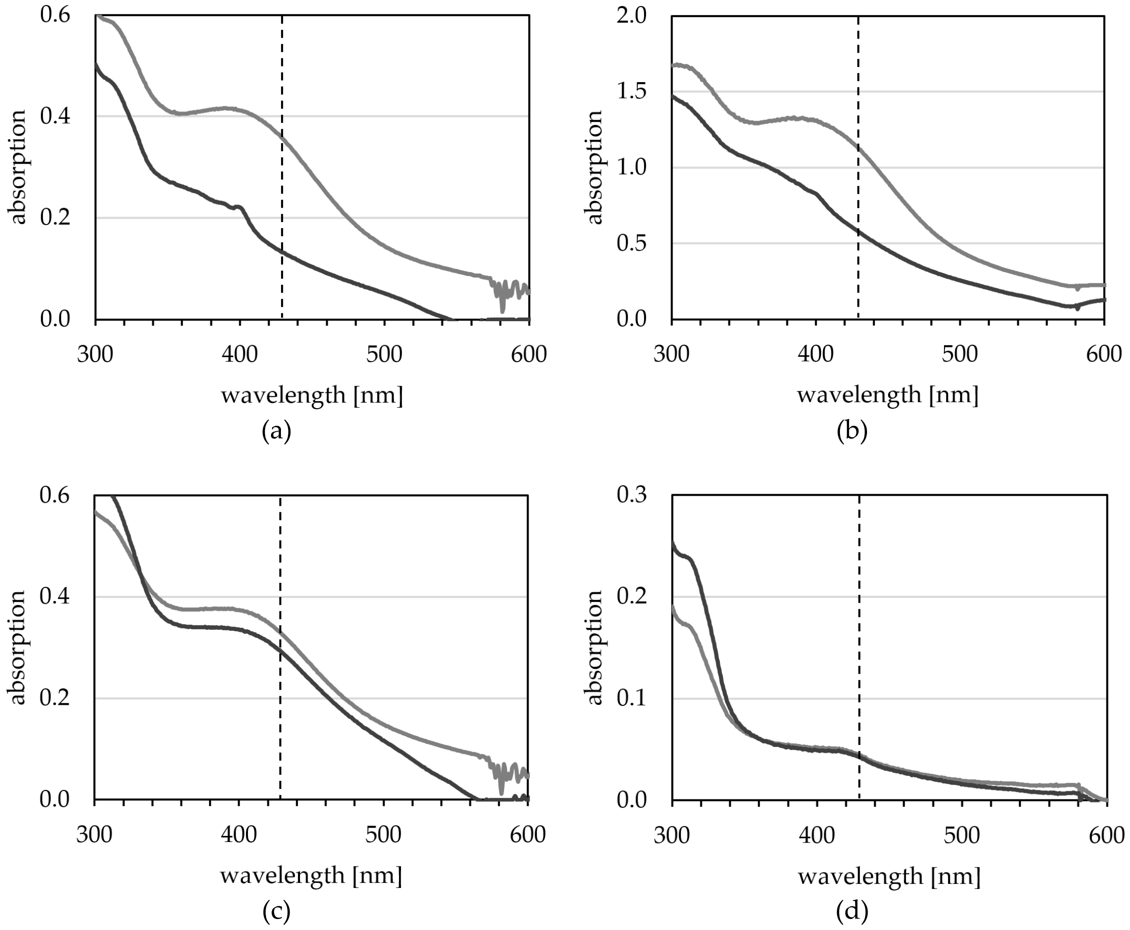

Figure S8. Fd reduction through AOR$_{X514}$. The spectrum was measured at 65 °C, in 50 mM TRIS between 300 and 600 nm after 27 µM His-Fd09620 (a), 29 µM His-Fd16450 (b), 27 µM His-Fd10420 (c) or 29 µM His-Fd19620 (d) was added (grey). The spectrum was measured again after 28 µg AOR-His and 3 mM acetaldehyde were added and the Fd reduction was followed at 430 nm (dark grey).

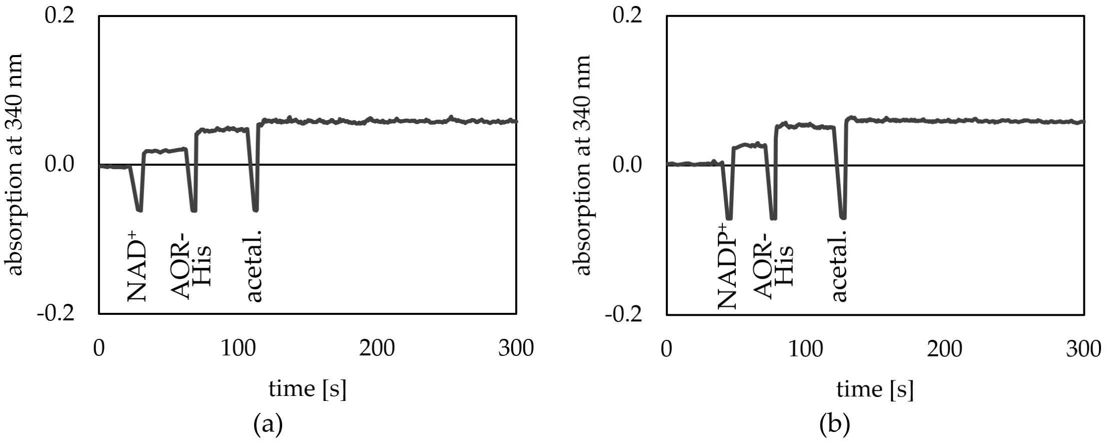

Figure S9. NAD(P)$^+$ dependent activity of AOR$_{X514}$. The activity was measured at 340 nM in 50 mM TRIS with 0.5 mM NAD$^+$ (a) or NADP$^+$ (b), 31 µg AOR-His and 1 mM acetaldehyde.

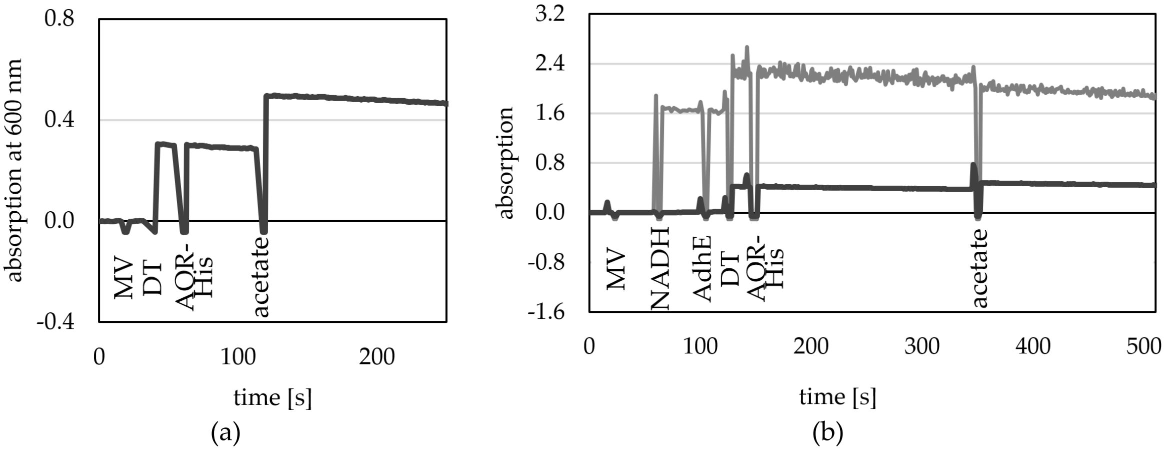

Figure S10. Acetate reduction activity assay of AOR_{X514} with MV and acetate. (a) An uncoupled assay was carried out at 65 °C in 50 mM TRIS buffer at pH 5.0, 10 µM MV was added and reduced with 1.8 mM dithionite (DT), 24 µg eluate from AOR_{X514} purification was added, the reaction was started with the addition of 230 or 460 mM acetate. MV oxidation was measured at 600 nm (ε_{MV}: 13.1 mM^{-1} cm^{-1}). (b) A coupled assay was carried out at 65 °C in 50 mM TRIS buffer at pH 5.0, 20 µM MV was added, 0.5 mM NADH and 15 µg AdhE-His (AdhE) were added and MV was reduced with 1.8 mM dithionite (DT), 60 µg eluate from AOR_{X514} purification was added, the reaction was started with the addition of 450 mM acetate. MV oxidation was measured at 600 nm (ε_{MV}: 13.1 mM^{-1} cm^{-1}; grey) and NADH oxidation at 340 nm (ε_{NADH}: 6.3 mM^{-1} cm^{-1}, dark grey).

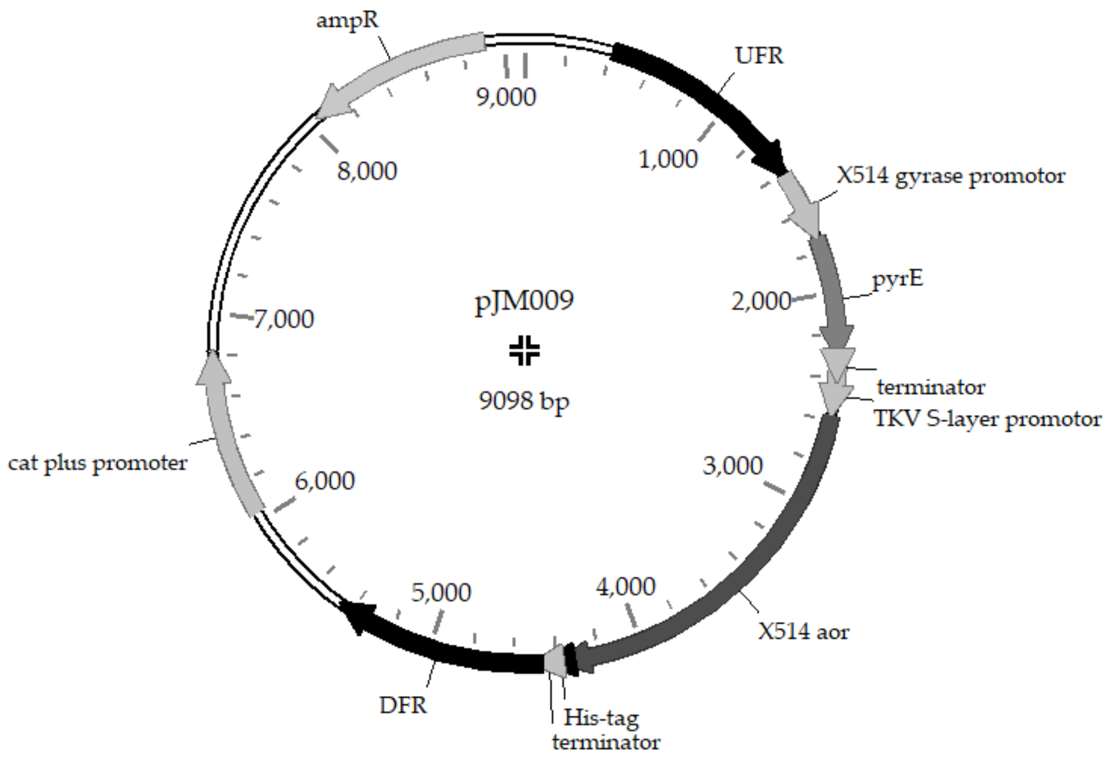

(a)

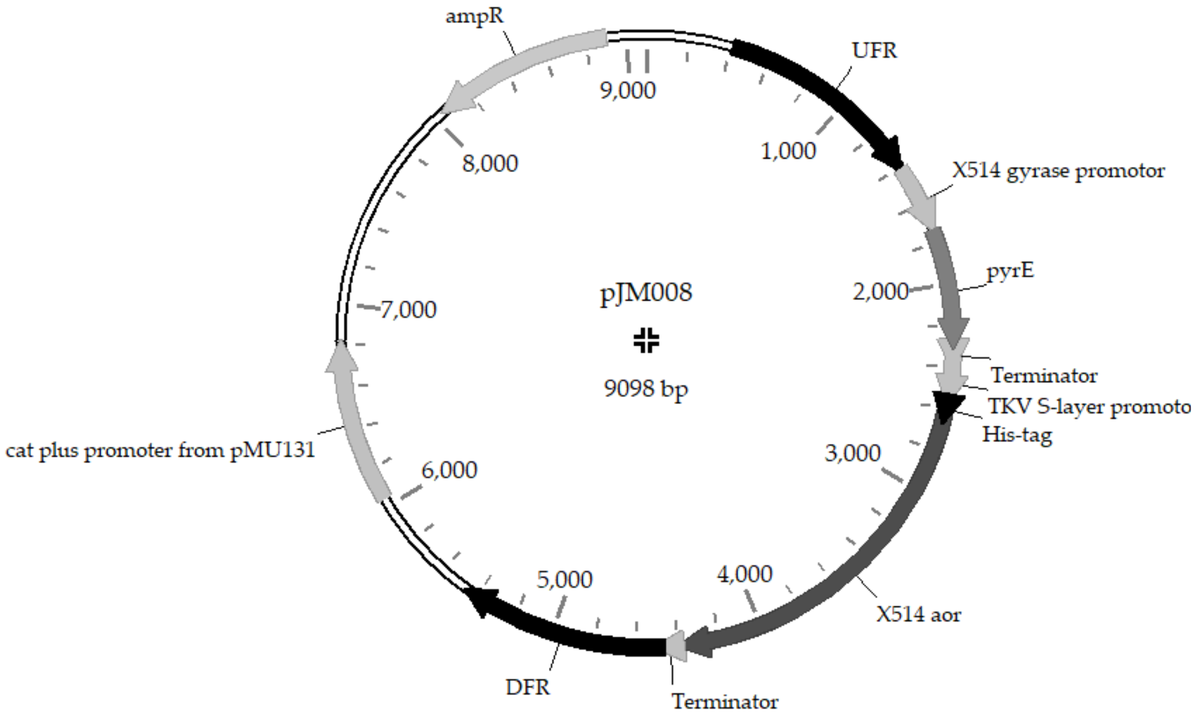

(b)

Figure S11. Plasmids pJM009 and pJM008. (a) Plasmid pJM009, used for the integration of *aor*-His into the genome of *T. kivui* and thereby generation of *T. kivui* MB014. (b) Plasmid pJM008, used for the integration of His-*aor* into the genome of *T. kivui* and thereby generation of *T. kivui* MB009.
